# Impaired retinoic acid signaling in cerebral cavernous malformations

**DOI:** 10.1038/s41598-023-31905-0

**Published:** 2023-04-05

**Authors:** Nastasja Grdseloff, Gwenola Boulday, Claudia J. Rödel, Cécile Otten, Daphné Raphaelle Vannier, Cécile Cardoso, Eva Faurobert, Deepika Dogra, Elisabeth Tournier-Lasserve, Salim Abdelilah-Seyfried

**Affiliations:** 1 Institute of Biochemistry and Biology, Department of Zoophysiology, University of Potsdam, Karl-Liebknecht-Strasse 24-25, 14476 Potsdam, Germany; 2 InsermNeuroDiderot, Université Paris Cité, 75019 Paris, France; 3 Department of Medical Genetics, Cumming School of Medicine, University of Calgary, Calgary, AB T2N 4N1 Canada; 4 Institute for Advanced Biosciences, INSERM 1209 CNRS, University Grenoble Alpes, 5309 Grenoble, France; 5 Institut Ruđer Bošković, Bijenička cesta 54, 10000 Zagreb, Croatia; 6 Service de Génétique Neurovasculaire, AP-HP, Hôpital Saint-Louis, 75010 Paris, France; 7 Institute of Molecular Biology, Hannover Medical School, Hannover, Germany

**Keywords:** Developmental biology, Molecular medicine

## Abstract

The capillary-venous pathology cerebral cavernous malformation (CCM) is caused by loss of CCM1/Krev interaction trapped protein 1 (KRIT1), CCM2/MGC4607, or CCM3/PDCD10 in some endothelial cells. Mutations of *CCM* genes within the brain vasculature can lead to recurrent cerebral hemorrhages. Pharmacological treatment options are urgently needed when lesions are located in deeply-seated and in-operable regions of the central nervous system. Previous pharmacological suppression screens in disease models of CCM led to the discovery that treatment with retinoic acid improved CCM phenotypes. This finding raised a need to investigate the involvement of retinoic acid in CCM and test whether it has a curative effect in preclinical mouse models. Here, we show that components of the retinoic acid synthesis and degradation pathway are transcriptionally misregulated across disease models of CCM. We complemented this analysis by pharmacologically modifying retinoic acid levels in zebrafish and human endothelial cell models of CCM, and in acute and chronic mouse models of CCM. Our pharmacological intervention studies in CCM2-depleted human umbilical vein endothelial cells (HUVECs) and *krit1* mutant zebrafish showed positive effects when retinoic acid levels were increased. However, therapeutic approaches to prevent the development of vascular lesions in adult chronic murine models of CCM were drug regiment-sensitive, possibly due to adverse developmental effects of this hormone. A treatment with high doses of retinoic acid even worsened CCM lesions in an adult chronic murine model of CCM. This study provides evidence that retinoic acid signaling is impaired in the CCM pathophysiology and suggests that modification of retinoic acid levels can alleviate CCM phenotypes.

## Introduction

Cerebral cavernous malformations (CCMs) are pathologies of the brain vasculature characterized by capillary-venous angiomas and recurrent bleedings. Genetic studies of familial forms of CCM showed that vascular lesions occur when an inherited heterozygous loss-of-function germline mutation is followed by a second somatic mutation affecting the remaining wild-type allele in some ECs in the central nervous system (reviewed in^1^). Currently, surgical treatment options are limited when such CCM lesions are positioned deeply within the brain or spinal cord. This has raised a strong interest in pharmacological interventions with the aim of (1) causing a regression or (2) prevention of bleeding of preexisting lesions. Previously, we performed a repurposed drug screen for compounds that prevented the appearance of *CCM* mutant phenotypes in zebrafish and the nematode *C. elegans*^2^. This led to the discovery that compounds targeting the retinoic acid (RA) pathway prevented CCM phenotypes in different animal models. Understanding whether RA signaling is affected in the CCM pathology may provide novel approaches for therapy.

**Figure 1:**
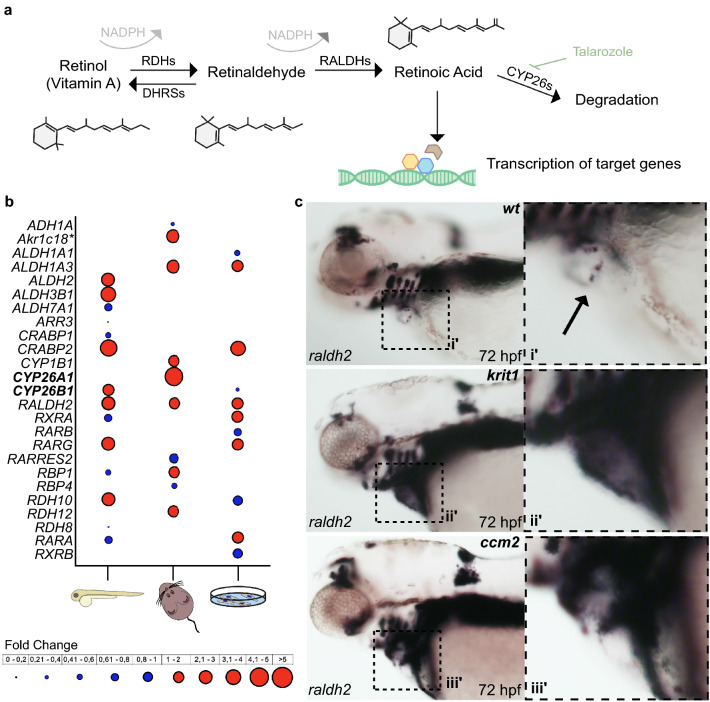
Expression of retinoic acid pathway components is misregulated in in vitro and in vivo models of CCM. **(a**) Retinoic acid synthesis and degradation pathway. Synthesis of retinoic acid from Vitamin A (retinol) is dependent on availability of NADPH. In this study, all-trans RA and the Cyp26 inhibitor Talarozole were used to modulate RA levels. (**b**) Comparative expression levels of RA pathway genes in *ccm2* mutant zebrafish hearts, pan-endothelial *Ccm2* knock-out mouse veins, and siRNA CCM2-depleted HUVECs. Fold changes in expression levels are depicted in circle sizes (grouped by fold changes between 0 and 5, see legend). Downregulated transcripts are represented with blue circles whereas upregulated genes are shown in red circles. Transcripts not represented in the datasets do not contain a circle. Gene names are represented as human orthologs except for genes for which only co-orthologs are known (marked with asterisks). (**c**) Whole-mount in situ hybridization of *raldh2* performed on 72 hpf old zebrafish and magnifications of the heart region (see box). Stained valve leaflets are marked by an arrow.

RA is a hormone derived from Vitamin A (Fig. 1a) that binds to nuclear receptors^3,4^ and changes their activities from repressors to activators. Within the vasculature, RA signaling promotes the maturation of endothelial cells (ECs) and has anti-proliferative effects during development and homeostasis of blood vessels^5,6^. In mice, loss of the main RA synthesizing enzyme Retinal dehydrogenase 2 (Raldh2, encoded by the *Aldh1a2* gene) causes major defects in embryonic vasculogenesis associated with increased proliferation and decreased maturation of ECs^6^. Molecular characterization of RA signaling targets in Raldh2-deficient mice uncovered a reduced expression of integrin α5, which is a transmembrane signaling protein with a limiting effect on EC proliferation^5^. Within the vasculature of the brain and spinal cord, RA signaling has a strengthening effect on cellular barriers. This has been demonstrated in murine, human, or human iPSC-derived ECs under in-vitro culture conditions^7–11^ and in animal models after ischemia^12^ or upon spinal cord injury^13^. Similarly, RA is required for integrity of the blood-retinal barrier in zebrafish^14^.

Modeling the CCM pathology in animal models has been a major challenge due to the chronic nature of the pathology in patients. Currently, we do not have one animal model available that can address all aspects of the CCM pathology. Instead, we need to use combinations of different models. Pan-endothelial knockouts in mice^15–20^ or complete loss of CCMs in zebrafish^21^ can model acute phases of bleeding and are important for elucidating the molecular and cellular mechanisms involved in CCM. On the other hand, chronic mouse models based on brain endothelial-cell-specific ablation of *Ccm* genes facilitate longitudinal observations of lesion formation and progression in CCM^22,23^.

While the physiological and developmental role of RA signaling in ECs is emerging, we still lack an understanding of its role under pathophysiological conditions. Here, we report on how changes in RA levels affect the severity of CCM phenotypes in animal models and human endothelial cells. We find that inhibition of RA degradation by Cyp26 or addition of moderate levels of RA alleviates CCM phenotypes. This finding may open new avenues towards therapeutic interventions.

## Materials and methods

### Mouse handling and strains

Mouse experiments were reported in compliance with the ARRIVE guidelines. All experimental animal procedures were in full accordance with the European Directive 2010/63/UE regarding the protection of animals used for scientific purposes. Authorizations were obtained from the French Ministry of Research following approval from the “Lariboisiere-Villemin” Ethic Committee on animal testing (APAFIS#12084-201710261854702v3 and APAFIS#12112-2017102714278853v3).

The acute EC*iCcm2* and chronic BEC*iCcm2* mouse models were previously described^16,22^ and refer to *Ccm2* ablation using the *Cdh5*(PAC)CreER^T2^ ^24^ or the *Slco1c1*CreER^T2^ ^25^ inducer. In both models, gene ablation was performed using a single dose of tamoxifen intra-gastrically (20 µg/g) at P1.

### Zebrafish genetics and maintenance

Handling of zebrafish was done according to FELASA guidelines, in compliance with German and Brandenburg state law, carefully monitored by the local authority for animal protection (LUVG, Brandenburg, Germany; Animal protocol #2347-18-2015) and in full accordance with the European Directive 2010/63/UE regarding the protection of animals used for scientific purposes. The following zebrafish strains were maintained under standard conditions as previously described^26^: *krit1*^*ty219c*^ ^27^; *ccm2*^*m201*^ ^28^, *Tg(kdrl:EGFP)*^*s842*^ ^29^.

### Mouse treatments and lesion burden assessment

The preventive trial was performed in the EC*iCcm2* acute mouse model using a daily administration of 1 mg/kg all-trans RA (Sigma R2625) given intra-gastrically at P2 and P3 and orally from P4 to P8. Animals were randomly administered with RA or vehicle only (1% DMSO in oil). Curative trials were performed in the BEC*iCcm2* chronic mouse model using subcutaneous implantation of 3-weeks release pellets (Innovative Research of America, Sarasota, FL) as described previously^22^. Treatment was administered in 3 months old animals using two dosages: 1.5 mg or 10 mg containing pellets with a 21-days release, corresponding to an equivalent dose of ~ 3 mg/kg/day or ~ 20 mg/kg/day for a mouse of 24 g. Animals were randomly implanted with either RA or placebo pellets. Assessment of lesion burden was performed as described previously^22^. Results were analyzed in regards to: (1) lesion area (percentage of area analyzed affected by CCM lesions), (2) number of caverns. Lesions were counted and categorized according to their size (small: < 5000 µm^2^, medium: 5–10,000 µm^2^, or large: > 10,000 µm^2^). Results are expressed as relative to total area analyzed. To compare the lesion burden between placebo versus RA-treated mice for each category of lesions (small-sized, medium-sized and large-sized lesions), three successive two-tailed Student’s T-tests were performed, followed by a Bonferroni correction for multiple testings (3 tests, one per lesion category). Adjusted p-values < 0.05 were considered statistically significant.

### Pharmacological treatment of zebrafish

The day before treatment, zebrafish carrying *Tg(kdrl:EGFP)*^*s843*^ as well as *krit1*^*ty219c*^ or *ccm2*^*m201*^ were incrossed and eggs were kept at 23 °C overnight in order to slow down development. The next day, 16/18-somite-stage embryos were dechorionated and transferred to E3 medium (5 mM NaCl, 0.17 mM KCl, 0.33 mM CaCl_2_, and 0.33 mM MgSO_4_) containing 1-phenyl-2-thiourea (PTU) (Sigma-Aldrich) to inhibit pigmentation. For treatment, either 0.1 µM Retinoic Acid (#R2625, Sigma) or 1–10 µM Talarozole (14531-10, Hycultec GmbH) was added and controls were supplemented with the same volume of DMSO.

### Zebrafish immunohistochemistry, imaging, and endocardial cell counting

Whole-mount immunohistochemistry was performed on 48 hpf embryos fixed overnight at 4 °C using 4% PFA. Permeabilization was done using cold acetone for 10–15 min at −20 °C. This was followed by one hour of incubation in blocking solution (PBST with 1% DMSO and 5% NGS). Antibodies were diluted in PBST with 1% DMSO and 1% NGS, then incubated overnight. The primary mouse anti-Zn-8/Alcam (1:25, Developmental Studies Hybridoma Bank) antibody and the secondary antibody Alexa Fluor 633-conjugated goat anti-mouse (1:200, Thermo Fisher Scientific) were used to label the heart. DAPI (4,6-diamidino-2-phenylindole; Sigma; 1:1000 was added in order to visualize nuclei. Specimen were mounted in low melting agarose (850080, Biozym) on glass bottom dishes (81158, Ibidi).

Images were taken on LSM 780 or LSM 880 confocal microscopes (Zeiss) and endocardial cells were counted using Imaris V9.9.1 (Bitplane, UK) in 3D reconstructions of confocal microscopy z-stacks. Endocardial cell nuclei were visualized by co-localization of *Tg(kdrl:EGFP)*^*s843*^ and DAPI. Maximum intensity projections and processing of pictures was done using Fiji^30^. Statistical analysis of endocardial cell numbers using an unpaired, two-tailed Student’s T-test was performed with Prism7 (GraphPad). Means were considered statistically significantly when P < 0.05.

### Zebrafish whole-mount in situ hybridization and image acquisition

Zebrafish were collected at 72 hpf, fixed with 4% PFA for 1.5 h at room temperature, and incubated in 100% Methanol at −20 °C for at least 16 h. Dig-labelled anti-sense mRNA probes for whole-mount in situ hybridzation were generated from wild-type cDNA by PCR using a reverse primer containing the T7 promoter sequence. The following PCR primers were used for template generation (5′ to 3′):*raldh2-*F AACGGGGGAAGCT-ACTGTTCA;*raldh2-*R GTAATACGACTCACTATAGGGGATTATCTCC-TCCTTGGCGATGC,*klf2a-F*: ATGGCAGGCGACTACAGAATG-3′;*klf2a-*R: TGTAATACGACTCACTATAGGGGATCAATGATAGGGCTTCTCGCC;

In vitro transcription was done using the T7 polymerase and the DIG RNA Labeling Kit (Roche). In vitro transcription was done using the T7 polymerase and the DIG RNA Labeling Kit (Roche). In situ hybridization was performed as previously described^31^, with the exception of probe hybridization at 67 °C. Stained embryos were mounted in Permount^32^ and images were acquired using an Axioskop microscope (Zeiss) and an EOS 5DMark III camera (Canon). Image processing was done with Fiji and Affinity Designer (Serif Europe).

### HUVEC culture, retinoic acid treatment and immunofluorescence staining

Pooled HUVECs (human umbilical vein endothelial cells) were obtained from Lonza and expanded over two passages in collagen 1 (from rat tail, BD) coated flasks in complete EGM-2 medium supplemented with 100 U/ml penicillin and 100 μg/ml streptomycin at 37 °C in a 5% CO_2_–3% O_2_ humidified chamber. HUVECs were transfected twice at 24 h-interval with 30 nM siRNA (Dharmacon smartpool ON-TARGET plus Thermo Scientific; Non-targeting siRNA #1, CCM2 ref. L-014728-01) and Lipofectamine RNAi max (Life Technologies, ref. 13778-150) according to the manufacturer’s instructions at 37 °C in a regular 5% CO_2_ in a humidified chamber. The day after transfection, HUVECs were seeded at 1.5 × 10^5^ cells in 24-well plates on coverslips coated with 10 μg/ml fibronectin (F0895, Sigma Aldrich) and incubated for 48 h total in complete EBM-2 medium supplemented with increasing concentrations of RA (Sigma Aldrich) with one renewal after 24 h. Cells were fixed with 4% PFA, permeabilized with 0.2% Triton X-100, and incubated with anti-beta-catenin mouse monoclonal antibody (6F9 clone, Sigma Aldrich). After rinsing, coverslips were incubated in Goat anti-Mouse AF 488 (Invitrogen, 1:1000) and phalloidin conjugates with TRITC (Sigma, 1:2000). The coverslips were mounted in Mowiol/DAPI solution and imaged on an epifluorescent Axio Imager microscope (Zeiss) at 63× magnification.

### Assesment of KLF2/4 expression levels by qRT-PCR

Total RNAs were extracted from mouse tissues and HUVECs using NucleoSpin RNA kits (Macherey–Nagel), including on-column DNase digestion. Reverse-transcription of mouse RNA was performed on 1 µg RNA using the Maxima first-strand cDNA synthesis kit (Thermo Scientific). Real-time PCR was performed on a LightCycler^®^ 480 (Roche) using the iQ™ SYBR Green Supermix (Biorad). Analysis was performed using the 2-DDCp method using *Gapdh* and *Pgk1* as housekeeping genes. The following primers were used (5′ to 3′):*Gapdh*-F: AATGTGTCCGTCGTGGATCT;*Gapdh*–R: CATCGAAGGTGGAAGAGTGG;*Pgk1*–F: CATAGGTGGTGGAGACACTGC;*Pgk1*–R: GTGCTCACATGGCTGACTT;*Klf2*–F: CGGCAAGACCTACACCAAG;*Klf2*–R: GGTAGTGGCGGGTAAGCTC;*Klf4*–F: AAGAACAGCCACCCACACTT;*Klf4*–R: GGTAAGGTTTCTCGCCTGTG.

RNA from HUVECs was reverse transcribed using iScript Reverse Transcription Supermix (BIO-RAD). Quantitative real-time PCR was performed with iTaq™ Universal SYBR Green Supermix (Promega) in a 25 µl reaction on a C-1000 Touch Thermal Cycler (BIORAD). Product sizes were controlled by DNA gel electrophoresis and the melt curves were evaluated using the BioRad CFX Manager. Ct-values were determined with the same software, and normalization was done with the house keeping genes GAPDH, RELA or ATP50, yielding comparable results. Expression levels of each target gene in the siRNA cells were calculated with ATP50 as reference gene and compared to CT. The following primers were used (5′ to 3′):KLF2-F: CATCTGAAGGCGCATCTG;*KLF2*-R: CGTGTGCTTTCGGTAGTGG;*KLF4*-F: TCTGATTACCCGGGCTGC;*KLF4*-R: GCGGTGCCCCGTGTGTTT;*ATP50-*F: ATTGAAGGTCGCTATGCCACAG;*ATP50-*R: AACAGAAGCAGCCACTTTGGG;*GAPDH*-F: ATCAGCAATGCCTCCTGCAC;*GAPDH*-R: AGTCTTCTGGGTGGCAGTGATG;*RELA*-F: CGGGATGGCTTCTATGAGG;*RELA*-R: CTCCAGGTCCCGCTTCTT.

For statistical analysis of KLF2/4 levels in HUVECs and mice, two-tailed unpaired Student’s T-tests were used to statistically compare differences in lesion area between the two groups of placebo versus treated animals. *P*-values < 0.05 were considered statistically significant.

### Transcriptomic analysis

Transcriptomic datasets were used from all three CCM models. Veins of post-natal day 15 (P15) EC*iCcm2* mice were extracted and used for transcriptome analysis^33^. Enrichment analysis was performed using a hypergeometric enrichment test for deregulation of genes belonging to the GO term Go:0001525 “Retinoid Metabolic process” (Enrichment = 2.15). Significance of the test was based on a hypergeometric p-value (Hypergeometric P-value = 0.004). Based on the differentially expressed genes in mice, we data mined the published CCM2-knock-out zebrafish dataset, which was based on whole heart tissue^34^. The same was done for *siCCM2*-treated HUVECs, for which transcriptomic data had recently been published^35^. Fold changes of genes of interest were visualized by grouped sizes of circles and plotted on a chart in an alphabetical order. Upregulated genes are shown in red, while downregulated genes are blue. Blank spaces without circles indicate that the gene was not represented in the transcriptomic dataset.

## Results

### Retinoic acid signaling is dysregulated in zebrafish, murine and HUVEC models of CCM

In a previous study we discovered that targeting the retinoic acid pathway pharmacologically in different models of CCM resulted in a suppression of the characteristic phenotypes^2^. In zebrafish, *ccm2*^*m201*^ mutant embryos showed endocardial over-proliferation and cardiac ballooning defects^34^. Treatment with either 13-cis-retinoic acid or all-trans retinoic acid between 18 and 48 h post fertilization (hpf) prevented this phenotype^2^. Positive effects of 13-cis-retinoic acid were also observed in ameliorating cellular phenotypes in human umbilical vein ECs (HUVECs) silenced with *CCM2* shRNA^2^. This suggested that changes in the levels of RA are causative to CCM phenotypes. To elucidate whether a loss of CCM proteins alters the RA synthetic and/or degradation pathway, we systematically analyzed the expression levels of genes encoding RA pathway components in CCM models in zebrafish, mouse, and HUVECs.

The RA synthetic pathway involves two enzymatic steps (Fig. 1a). Retinol is first converted into retinaldehyde by cytosolic alcohol dehydrogenases and then oxidized to RA mainly by Raldh2^36^. In a previous microarray study of the 72 hpf *ccm2*^*m201*^ mutant zebrafish endocardial and myocardial transcriptome^34^, genes encoding the major RA synthetic enzymes such as *Raldh2* were strongly elevated. In line with this data, analysis of transcriptomic microarray data from pan-endothelial *Ccm2* murine knockout mice (EC*iCcm2*)^33^ showed an enrichment of deregulated genes related to the gene ontology (GO) term “Retinoid Metabolic Process” (Enrichment = 2.15; hypergeometric p-value = 0.004). In this dataset, RA synthesis enzymes including *Raldh2* and *Retinol dehydrogenase 12* (Fig. 1b) were both upregulated. These findings were also confirmed by transcriptomic data from *siCCM2*-treated HUVECs^35^. Interestingly, another common finding in all three CCM models was the simultaneous upregulation of genes encoding RA degrading enzymes Cyp26A or Cyp26B (Fig. 1b).

Next, we performed whole-mount in situ hybridizations against *raldh2* mRNA to clarify whether its expression was higher within the endocardium of *ccm2*^*m201*^ and *krit1*^*ty219c*^ zebrafish mutants. This was indeed the case at 72 hpf (Fig. 1c), whereas wild-type embryos had only an expression domain at the atrioventricular canal (see arrow Fig. 1c). These findings demonstrated that a loss of CCM proteins causes an increased expression of RA pathway genes.

### Pharmacological intervention of the retinoic acid degradation pathway impacts endocardial cell numbers in *krit1* zebrafish mutants

In our previous study, we found that the treatment of *ccm2* mutant zebrafish with 13-cis-RA and all-trans RA caused a phenotypic rescue^2^. This raised the possibility that RA levels are actually lower in CCM despite an upregulation of RA pathway genes. In support of our hypothesis, zebrafish *raldh2* mutants, which lack RA, have enlarged hearts due to an over-proliferation of cardiac cells^37,38^, thus resemble the *ccm* mutant cardiac ballooning phenotype^34^. This prompted us to perform further pharmacological studies in zebrafish, HUVECs and mice with the aim to elevate RA levels in CCM models.

**Figure 2:**
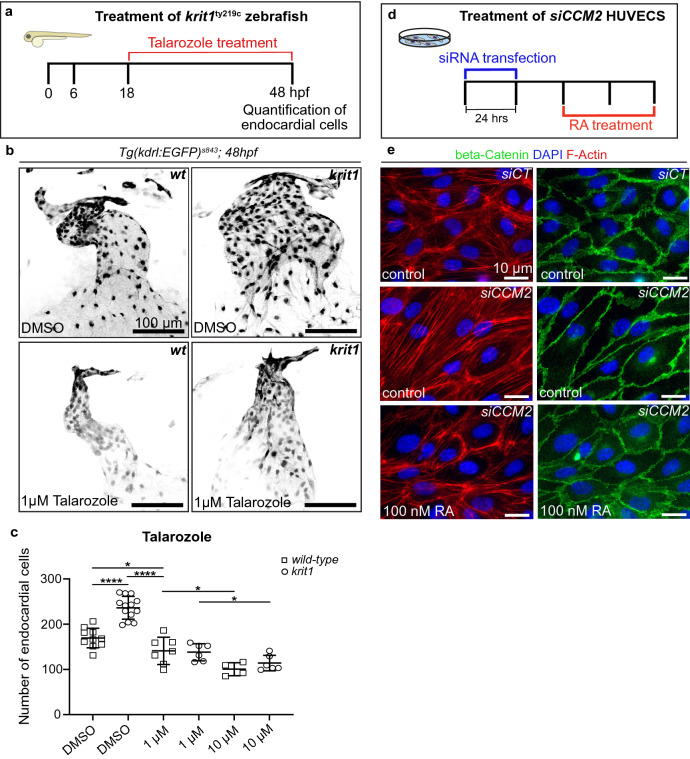
Curative effects upon Talarozole treatment of *krit1* mutant zebrafish and retinoic acid treatment of *siCCM2* depleted HUVECs. **(a**) Treatment scheme for zebrafish from 18 to 48 hpf using Talarozole. Endocardial cell numbers (a readout for CCM-associated cardiac phenotype in zebrafish) were analyzed after treatment in two independent experiments. (**b**) Representative images of zebrafish heart morphologies visualized by the transgene *kdrl:EGFP* at 48 hpf after treatment. Upper: heart morphology of controls treated with DMSO. Lower: hearts treated with Talarozole. Scale bars: 100 µm. (**c**) Endocardial cell counts at 48 hpf of zebrafish treated with several doses of Talarozole or DMSO (*P < 0.05; ****P < 0.0001 vs. control). Each data point represents one heart. (**d**) Treatment regimen of HUVECS. SiRNA transfection was done for 24 h, followed by treatment with RA 24 h later. Cell morphologies were analyzed after 48 h of incubation and antibody staining. (**e**) Immunohistochemistry of HUVECS for beta-catenin, F-actin, and DAPI. First column: control cells (siCT) with wild-type *CCM2* expression. Second column: untreated *siCCM2*-depleted HUVECs. Third column: representative image showing *siCCM2* HUVECs treated with 100 nM RA. Scale bars: 10 µm.

For achieving this goal, we used an inhibitor of the RA degrading enzyme Cyp26 (Talarozole) and treated *krit1*^*ty219c*^ mutant zebrafish from 18 to 48 hpf at a concentration of 1 or 10 µM (Fig. 2a). We found that this treatment was effective because there was a reduction of the zebrafish ballooning heart phenotype (Fig. 2b) and a significant reduction of endocardial cell numbers in *krit1*^*ty219c*^ mutants (P ≤ 0.0001) (Fig. 2c). This finding was in strong support of our hypothesis that increasing RA levels by preventing its degradation suppresses CCM phenotypes.

### Elevation of retinoic acid levels in si*CCM2* HUVECs has dose-dependent rescue effects

In HUVECs, characteristic CCM phenotypes include the formation of actin stress fibers and a massive cell elongation (Fig. 2e). Here, we assessed the effects of RA treatment on si*CCM2* HUVECs. We treated these cells with 100 nM RA for a period of 48 h (Figs. 2d; S1a,b). Then we stained F-actin within the cells and performed immunohistochemistry against beta-catenin. These stainings revealed that treatment with RA caused a reduction of actin stress fibers and a rescue of cell sizes in HUVECs (Fig. 2e). The same CCM phenotypes were not rescued when we exposed HUVECs to higher or lower doses of RA in the range of 50–200 nM (Fig. S1b). This suggested that RA has highly dosage-sensitive effects with an optimal dose at 100 nM in preventing CCM phenotypes in HUVECs. In a separate experiment, we treated *krit1*^*ty219c*^ mutant zebrafish with RA at a dose of 0.1 µM. This treatment caused a drastic effect on heart morphology and demonstrated that ectopic addition of RA has a strong impact on the zebrafish heart phenotype (P ≤ 0.0001) (Fig. S1c,d).

### Ectopic administration of RA lacks preventive and curative effects in preclinical trials with mouse models of CCM

Subsequently, we performed preclinical trials using *CCM2*-deficient mice and assessed the effectiveness of retinoic acid treatment in these models. Mouse models have become important tools in preclinical drug trials against CCM^2,18,19,22,39–45^. First, we performed a preventive trial in the acute pan-endothelial *iCcm2* knockout mouse (EC*iCcm2*)^16^ (supplemental Fig. S2a) with the aim to reduce or delay lesion formation. Upon tamoxifen-induced *Ccm2* gene ablation at postnatal day 1 (P1), pups developed high lesion burden mainly in the cerebellar and retinal vasculature by P8. We found that administering low daily doses of all-trans RA (1 mg/kg) from P2 (before onset of the disease) to P8 did not reduce their lesion burden at day P8 compared to vehicle treated pups (P > 0.05) (Fig. S2b). Importantly, we noticed that RA had a negative impact on the survival rate of pups (Fig. S2c). This may be due to developmental side effects caused by the RA treatment. Taken together, these findings suggest that, with the concentration used in this study, all-trans RA treatment does not prevent de novo formation of vascular lesions in new born mice. These results also highlighted the need for chronic mouse models with long-term survival and slower development of the disease, which targets adult stages to avoid detrimental developmental effects of RA treatment.

**Figure 3:**
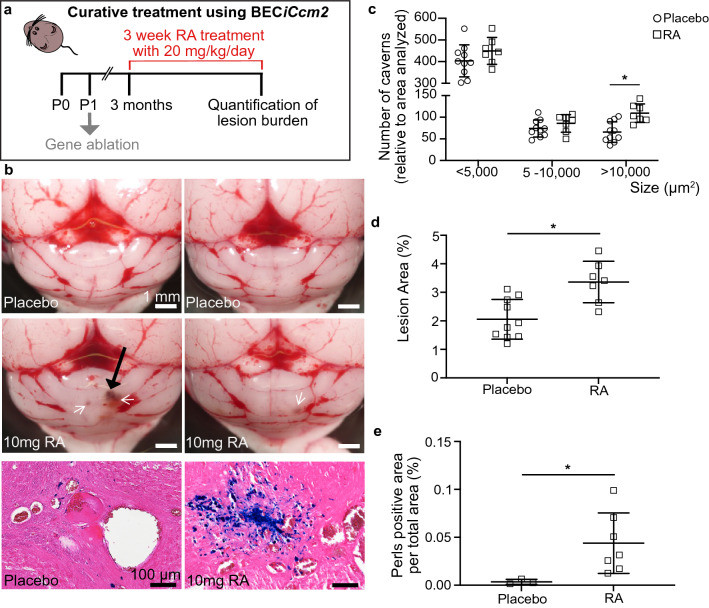
Curative RA trial in BEC*iCcm2* mice. **(a)** Treatment regimen with brain-endothelial-cell-specific gene ablation at P1 followed by RA treatment at three months of age for a period of 3 weeks (10 mg containing pellets with a release of ~ 20 mg/kg/day for 21 days). (**b)** Extracted BEC*iCcm2* mouse brains after treatment. Upper: placebo treated controls. Middle: brains after 10 mg of RA treatment. Black arrows indicating hemorrhage, white arrows pointing at visible lesions. Lower: histological sections of cerebral cavernous malformation (CCM) lesions after treatment. Perls blue stain reveals non-heme iron deposition. Scale bars: 1 mm (upper and middle); 100 µm (lower). (**c)** Quantification of lesion burden in cerebella of placebo versus 10 mg RA treated mice. Lesions are quantified in three groups according to their size. Each datapoint represents one animal. Error bars represent mean with SD. (**d**) Quantification of lesion area as percentage of total area analyzed (*P < 0.05). (**e**) Quantification of Perls blue staining positive area per total area analyzed (*P < 0.05; **P < 0.01). Error bars represent mean with SD.

Thus, we continued with a curative longitudinal trial of all-trans RA treatment in a chronic CCM mouse model with the aim of testing whether existing lesions could be reduced in size. For this trial, we used a mouse model with inducible brain-endothelial-cell-specific ablation of *Ccm2*^22^, using *Slco1c1*CreERT2, which is based on a thyroid hormone transporter expressed in endothelial cells of the blood–brain barrier (BEC*iCcm2*)^25^. The ablation of *Ccm2* was induced at P1 and the treatment was initiated at 3 months of age after CCM lesions had developed within the brain vasculature^22^. RA was administered via subcutaneous implantation of RA-containing pellets diffusing the compound for a period of 3 weeks (Fig. 3a and Fig. S2d). Non-treated BEC*iCcm2* were implanted with pellets containing placebo. We employed two doses of RA corresponding with a low dose of approximately 3 mg/kg/day and a high dose of approximately 20 mg/kg/day. No side effects were observed under either of these two treatment paradigms. To assess whether the administrated retinoic acid pellets were bioactive, we performed qRT-PCR analyses of several known RA target genes including *Cyp26b1,* which is expressed in the murine brain^46^ (Fig. S3). Treatment with a low dose of all-trans RA led to higher gene expression in a limited number of RA target genes but had no effect on cavernoma lesion burden or sizes (Figs. S2d–f and S3a). By contrast, treatment with the high dose of all-trans RA triggered higher expression levels of several RA target genes within the cerebellum, demonstrating effective bioactivity of all-trans RA from implanted pellets (Fig. S3b). Unexpectedly, under this high-dose RA treatment, the lesion burden increased with significantly higher numbers of large-sized lesions within cerebellar tissue (P < 0.05) (Fig. 3b–d) as well as increased cerebral hemorrhage in treated animals (P < 0.05) (Fig. 3e). This demonstrated that treatment with a high-dose of all-trans RA worsens CCM lesion formation in the chronic mouse model. Furthermore, it confirmed that RA signaling is involved in the pathological process of CCM development.

### RA treatment does not affect *KLF2/4* mRNA levels in si*CCM2* HUVECs and CCM animal models

**Figure 4:**
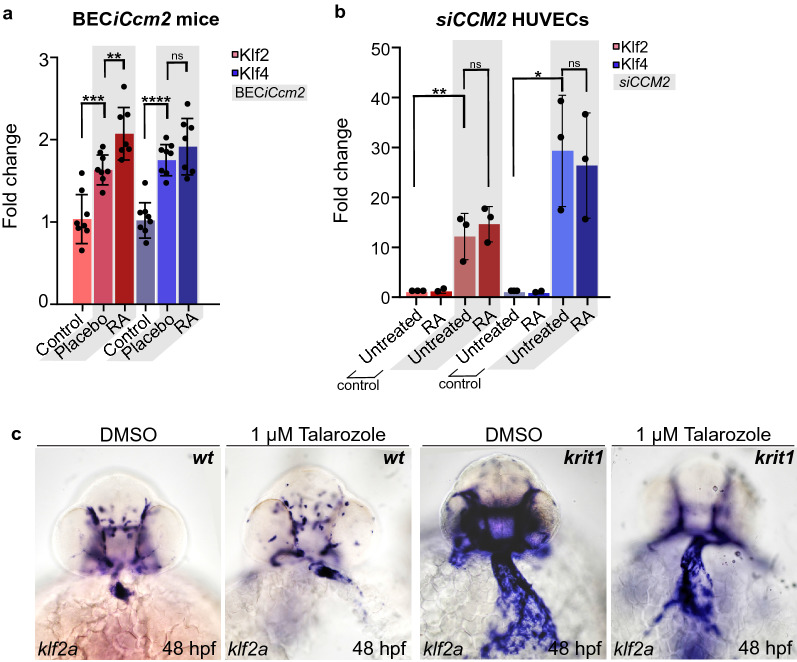
Assessment of *KLF2/4* mRNA expression in treated animal models of CCM. **(a**) Expression levels of treated BEC*iCcm2* mouse cerebella after curative RA treatment (~ 20 mg/kg/day for 21 days) as mean fold changes determined by qRT-PCR. (ns = P > 0.05; **P < 0.01;***P < 0.001;****P < 0.0001 vs. control). Each data point represents one sample. Error bars show mean with SD. (**b**) Fold changes of *KLF2/4* mRNA expression in *siCCM2*-transfected HUVECs treated with RA for 48 h compared to untreated cells (*P < 0.05; **P < 0.01). Each data point represents one sample. (**c**) Whole-mount in situ hybridization of *klf2a* in 48 hpf zebrafish embryos treated with DMSO or Talarozole.

In the CCM pathology, the two transcriptional regulators KLF2/4 become highly expressed within endothelial cells^18,20,34,47^. To assess whether RA treatment had an effect on *KLlf2/4* mRNA expression, we performed comparative qRT-PCR studies in CCM in murine and human endothelial cell models of CCM. First, we analyzed mouse cerebellar tissue, in the BEC*iCcm2* model and compared gene expression in treated animals and controls. This revealed that levels of *Klf2* transcripts were elevated upon (high dosage) RA treatment in mice (P < 0.01), and even higher than in untreated controls (Fig. 4a). However, in CCM2-deficient HUVECs, *KLF2/4* mRNA levels were not altered upon treatment with RA at a dose of 100 nM (Fig. 4b). Finally, we tested in zebrafish by whole-mount in situ hybridization experiments on *krit1*^*ty219c*^ mutants, whether Talarozole treatment normalized *klf2a* mRNA levels. Antisense probes against *klf2a* mRNA were used in zebrafish embryos that had been treated with 1 µM Talarozole (Fig. 4c). This treatment revealed that zebrafish embryos in which endocardial cell numbers had been reduced by Talarozole (Fig. 3c), expression levels of *klf2a* mRNA remained elevated. Hence, the curative effects of RA or Talarozole treatment do not involve a normalization of *KLF2/4* mRNA levels.

## Discussion

In this study, we show that the RA synthesis pathway is dysregulated in different animal models of CCM and in *CCM2*-deficient HUVECs. Our study explores curative approaches for CCM based on changes in RA levels. In particular, we find that inhibiting the RA degradation pathway by blocking Cyp26 had a restorative effect in zebrafish. This suggests a novel approach to alleviate CCM phenotypes. We also find that treating si*CCM2*-silenced HUVECs with RA rescued the endothelial cell morphology. Similarly, the *krit1* mutant zebrafish endocardial phenotype was reduced by increasing RA levels. However, treatment with low or high doses of all-trans RA-releasing pellets was not successful in a preclinical chronic mouse model of *Ccm2.* This could be due to the difficulties of restoring (1) physiological levels of RA and/or (2) RA levels specifically within CCM-deficient ECs. We observed that a high dose of 20 mg/kg/day even worsened lesion formation in this mouse model. Potentially, high RA levels may not only affect CCM-deficient ECs but may also impact the integrity of neighboring wild-type ECs.

RA has been used to strengthen EC junctional properties in different cell culture models of blood brain barrier formation^7,8,10,48,49^. A weakening of EC junctions is a hallmark feature of the CCM pathology^1^. However, the concentrations of RA needed in cell culture models to induce junctional barriers are much higher^7^ than the physiological RA levels detected within embryonic tissues^50^. Furthermore, increasing evidence suggests that RA produced by non-endothelial tissues such as the brain is required for junctional maturation and maintenance^8^. For instance, the endothelial-specific disruption of RA signaling did not affect the integrity of the blood brain barrier, unlike a general RA knockout that had a dramatic effect on junctional integrity^7,48^.

The curative effects of treating CCM-deficient zebrafish with RA or by inhibiting Cyp26 with Talarozole did not involve a reduction of elevated expression levels of *klf2a* mRNA. Similarly, treatment of CCM2-deficient HUVECs with 100 nM of RA alleviated the cellular phenotypes but had no effect on elevated *KLF2/4* mRNA levels. These findings suggest that pathways parallel to those regulated by the two transcriptional regulators KLF2/4 are involved in producing CCM phenotypes and that RA signaling is involved. It will be important to identify those pathways regulated by RA signaling. For instance, RA signaling has a stabilizing effect on cell junctions^7–11^ and prevents over-proliferation of ECs^5,6^, which are two processes that are impaired in CCM^21,34^. In the zebrafish, RA plays a role in blood-retinal barrier integrity^14^.

In our hands, curative RA treatment approaches may have been complicated by different dosage effects on the cerebral vasculature. There is evidence that regions of the brain vasculature that need to maintain weakened cell junctions have lower levels of RA activity. One such example is the hypothalamic-hypophyseal region of the zebrafish brain that plays a central role in the release of hormones into the blood. Here, the activity of RA is apparently reduced due to high expression levels of the RA-degrading enzyme Cyp26b within surrounding cells^51^. The inhibition of this enzyme caused higher expression of Claudin-5 and enhanced barrier properties of this vasculature. This study suggests that an alternative approach for strengthening barrier properties of CCM-deficient ECs may be inhibition of Cyp26b to prevent the local degradation and to reach physiological levels of RA.

Currently, we can only speculate about the reasons for a deregulated RA signaling in CCM. One hallmark feature of the CCM pathology has been increased oxidative stress^52^. This happens when free reactive oxygen species (ROS) such as superoxide anions and cellular antioxidants become unbalanced. KRIT1-deficient ECs have higher levels of NADPH oxidase-mediated redox signaling, which results in oxidative stress^53–55^. This causes a depletion of NADH, an essential co-factor of multiple metabolic and anabolic enzymes, including the RALDH family proteins that catalyze the synthesis of RA. Because expression of Raldh enzymes is negatively regulated by their biochemical endproduct RA^56^, high expression levels of *raldh2* may indicate subphysiological levels of RA in *ccm* mutant ECs. Pharmacological suppression screens revealed that the free radical scavenger tempol and antioxidants such as Vitamin D^2,40^ have a beneficial role in CCM. Interestingly, Vitamin D receptor (VDR) and retinoid X receptor (RXR) are nuclear receptors, which are involved in breast cancer treatment approaches^57^. Both receptors dimerize and activate common transcriptional targets^58,59^. Our current findings raise the possibility that Vitamin D and RA have joint roles also in suppressing CCM. The progesterone receptor is another breast cancer-relevant nuclear receptor and has been linked to CCM as well^60,61^. A disruption of the balance between CCMs and nuclear as well as membrane progesterone receptors may lead to increased EC and blood–brain-barrier permeability^61^. The link between progesterone and retinoic acid receptors has been established in the context of breast cancer^62^. While the commonalities of breast cancer and CCM remain to be better understood, increasing evidence is emerging that there are common roles of nuclear receptors and their targets in CCM.

Our results clearly point towards a role of retinoic acid in modulating the CCM phenotype. They indicate that a change in RA levels could be responsible for the increase in hemorrhages and lesion sizes observed in CCM. Even though pharmacological intervention appears very challenging, our findings may eventually come to benefit patients. Oral intake of vitamin A or application of RA-containing cosmetics on a regular basis could have an impact on disease progression. Further studies are needed to evaluate whether a cautious lifestyle, where RA levels are kept in balance, could be beneficial for patients.

## Supplementary Information

Supplementary Legends.

Supplementary Figure S1.

Supplementary Figure S2.

Supplementary Figure S3.

## Data Availability

The datasets used and/or analysed during the current study available from the corresponding author on request.
