# Supplementary material for: Impaired retinoic acid signaling in cerebral cavernous malformations
Source: Sci Rep. 2023 Apr 5;13:5572. doi: 10.1038/s41598-023-31905-0 (PMC10076292; doi:10.1038/s41598-023-31905-0)
Supplement: Supplementary file 1 — Supplementary Legends. [file 41598_2023_31905_MOESM1_ESM.pdf]

Supplementary Figures

Figure S1 Effects of non-optimal RA dosages on *siCCM2*-treated HUVECs and RA treatment of *ccm1* mutant zebrafish. a, Experimental outline of HUVEC treatments. SiRNA was transfected for 24 hrs and treatment started 24 hrs later. HUVECs were exposed to different concentrations of RA for 48 hrs. **b,** Immunohistochemistry against beta-Catenin, F-Actin, and DAPI of control and transfected cells. **c**, Quantification of endocardial cells in *ccm1* mutant zebrafish treated with retinoic acid and DMSO controls (*=P<0.05; ****=P<0.0001). Each data point represents one sample. Error bars represent mean with SD. **d**. Morphology of *ccm1* mutant zebrafish heart after treatment with 0.1 µM retinoic acid.

Figure S2 Preventive and low-dose curative trial of *Ccm2* mutant mice. A-C: Effect of RA evaluated in the rapid ECiCCM2 mouse model. **a,** Treatment scheme of the preventive treatment approach in the pan-endothelial gene ablation model. Mice were treated with a daily dose of 1 mg RA from P2 (before CCM phenotypes become apparent) to P7, the lesion burden was assessed at P8. **b**, Number of caverns found in the cerebellum in vehicle-treated control mice vs. mice treated with 1mg of RA. **c**, Comparative survival curve of wild-type and i*Ccm2*-mice undergoing the treatment regimen. Survival is shown as a percentage over time. **d-e**: Curative effects of RA in the chronic BEC*iCcm2* mouse model. **d**, Gene ablation was initiated at P1 and treatment started after three months (after the CCM phenotype has developed) for a duration of three weeks using a 1.5 mg RA pellet with a daily diffusion of ~3 mg/kg for a mouse of 24 g. **e**, Quantification of mouse cerebellar lesion area relative to the entire area analyzed in placebo- and RA-treated brains. **f**, Number of caverns analyzed in three

groups according to lesion size. Counts are normalized to the entire area analyzed. Error bars represent mean with SD.

Figure S3 Expression levels of retinoic acid-responsive genes after low- and high-dose RA treatment regimens in BEC*iCcm2* animals by qRT-PCR. a, Relative mRNA expression levels of changed transcripts in liver and cerebellar tissues of placebo vs. low-dose RA-treated samples (~3 mg/kg/day for 3 weeks). **b**, Cerebellar samples after high-dose RA treatment (~20 mg/kg/day for 3 weeks). Relative expression levels are shown as determined by qRT-PCR (*=P<0.05;**=P<0.01;***=P<0.001). Error bars represent mean with SEM.
