# Supplementary figures and images for: Impaired retinoic acid signaling in cerebral cavernous malformations
Source: Sci Rep. 2023 Apr 5;13:5572. doi: 10.1038/s41598-023-31905-0 (PMC10076292; doi:10.1038/s41598-023-31905-0)

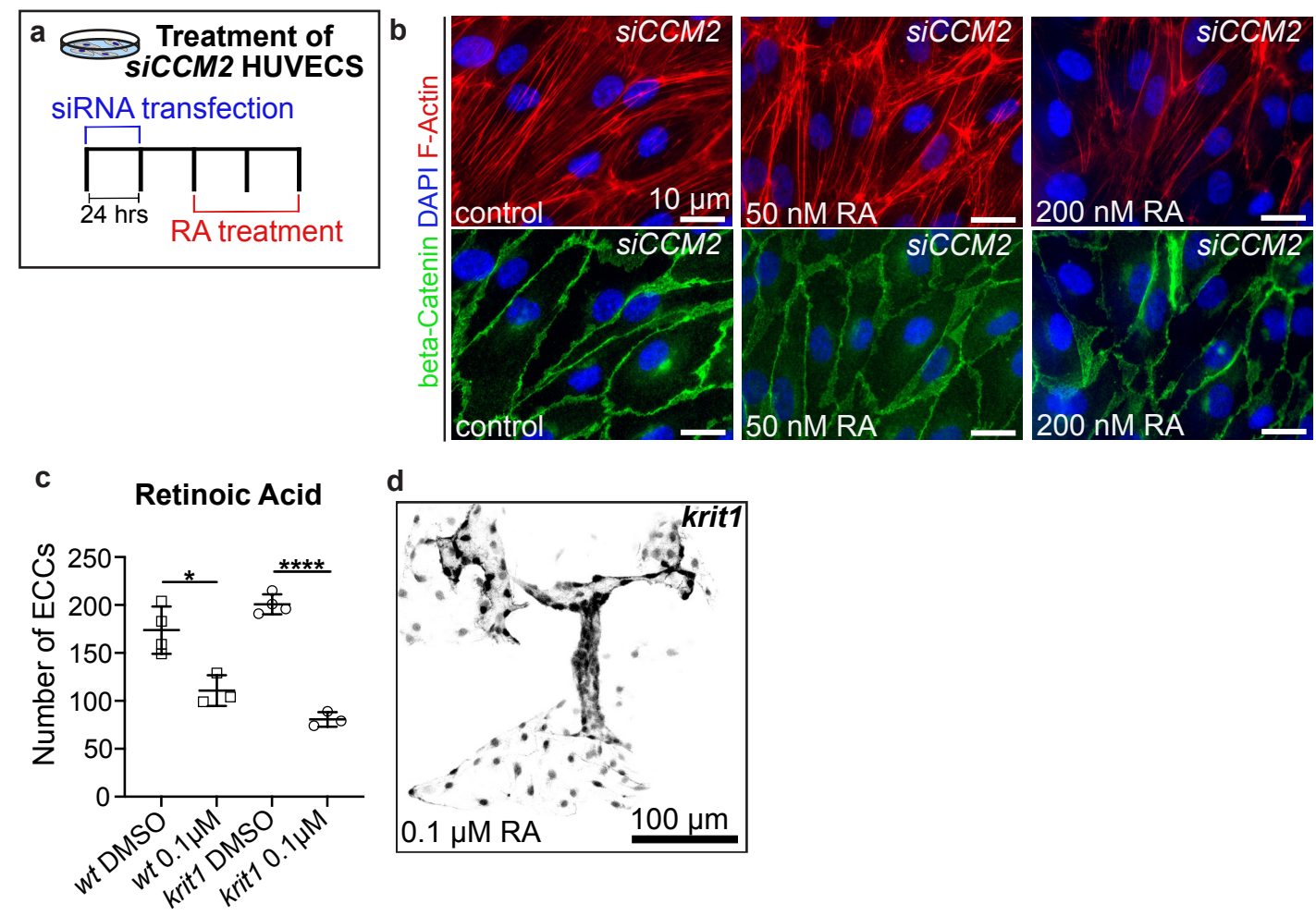

Supplement: Supplementary file 2 — Supplementary Figure S1. [file 41598_2023_31905_MOESM2_ESM.pdf]

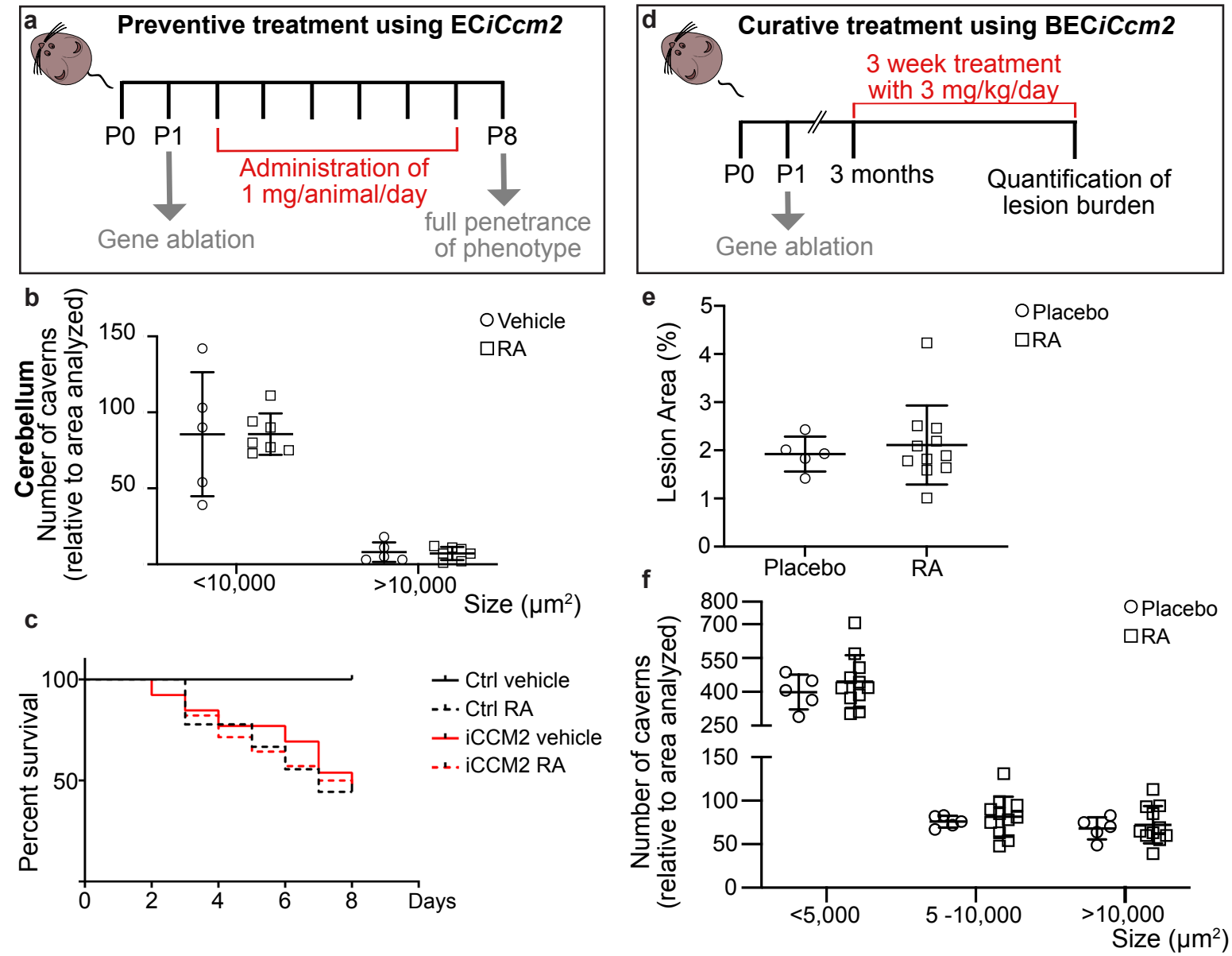

Supplement: Supplementary file 3 — Supplementary Figure S2. [file 41598_2023_31905_MOESM3_ESM.pdf]

**a**

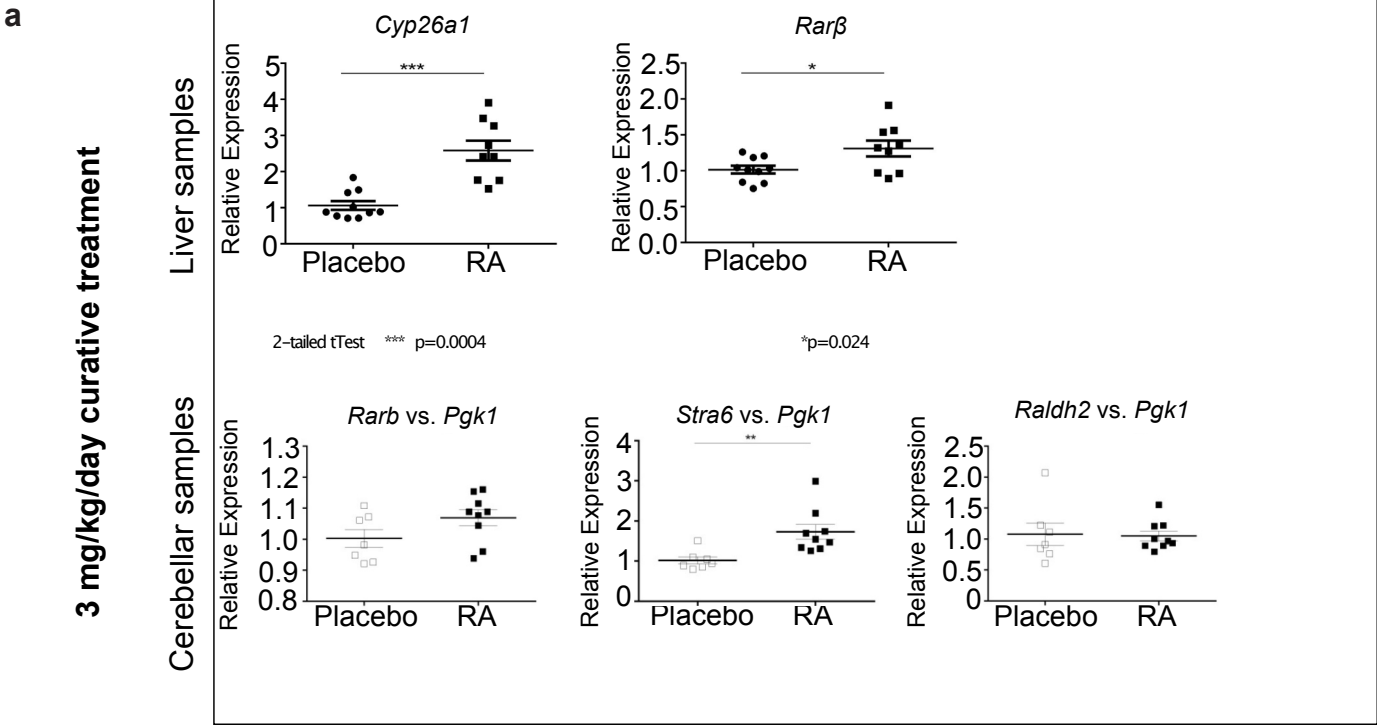

**b**

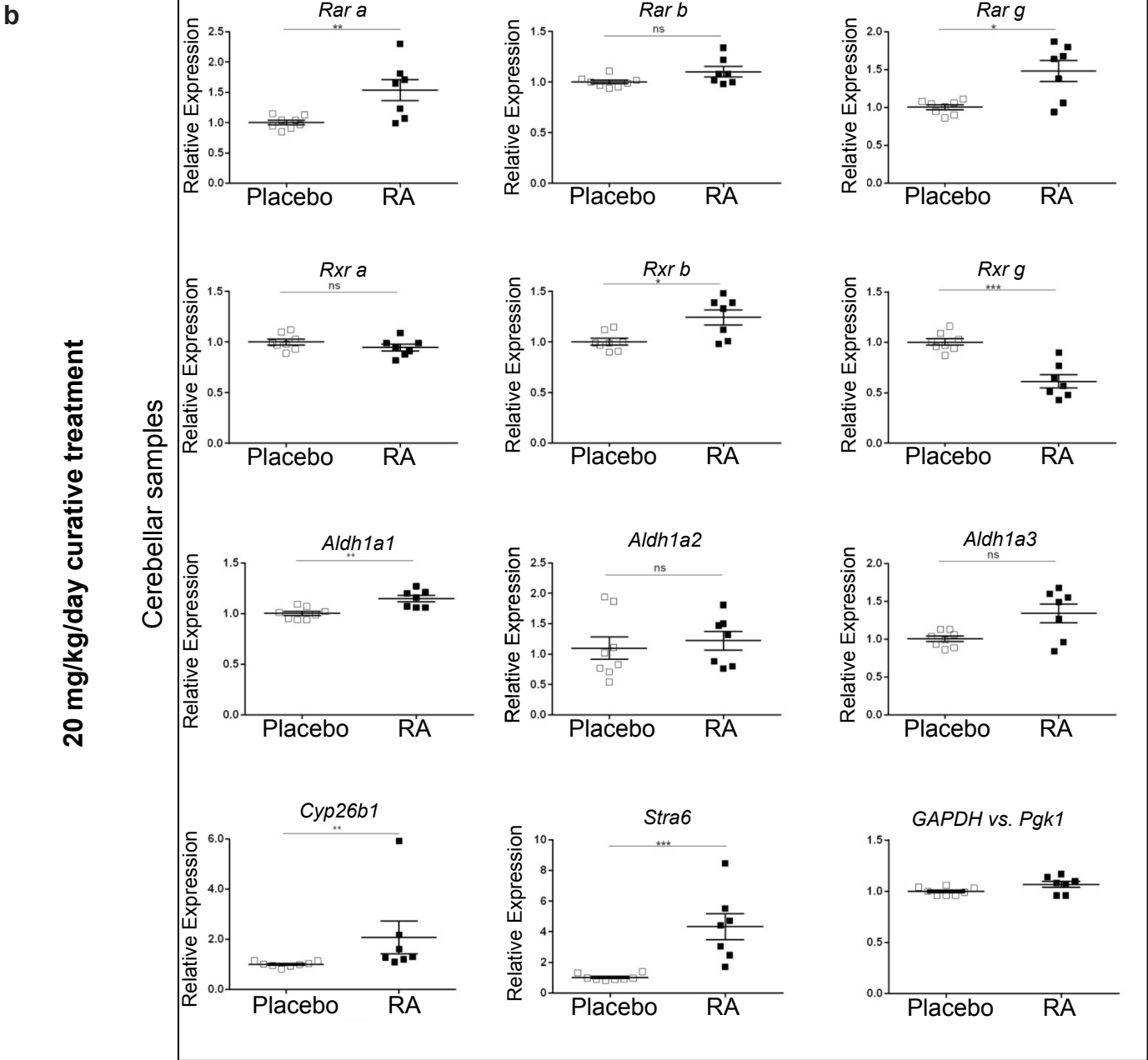

Supplement: Supplementary file 4 — Supplementary Figure S3. [file 41598_2023_31905_MOESM4_ESM.pdf]
